# Impact of Zinc(II) Chloride Contamination on Bentonites: Formation of Simonkolleite and Effects on Porosity and Chemical Composition

**DOI:** 10.3390/ma18132933

**Published:** 2025-06-20

**Authors:** Edyta Nartowska, Piotr Stępień, Maria Kanuchova

**Affiliations:** 1 Faculty of Environmental Engineering, Geodesy and Renewable Energy, Kielce University of Technology, 25-314 Kielce, Poland; 2 Faculty of Civil Engineering and Architecture, Kielce University of Technology, 25-314 Kielce, Poland; pstepien@tu.kielce.pl; 3 Faculty of Mining, Ecology, Process Control and Geotechnologies, Technical University of Kosice, Letna 9, 042 00 Kosice, Slovakia; maria.kanuchova@tuke.sk

**Keywords:** clay minerals, porous materials, sealing materials, bentonite, zinc(II) chloride, porosity, specific surface area, geotechnical properties

## Abstract

This study examines the formation of the clay mineral simonkolleite (Skl) in bentonites contaminated with zinc(II) chloride (ZnCl_2_), a process that has been little documented in heterogeneous systems such as contaminated bentonites. We explain the contamination mechanisms and provide new insights into the mineralogical, structural, and physicochemical transformations occurring within these materials. The objective, explored for the first time, was to assess how the ZnCl_2_-induced mineral phase formation influences the properties of bentonites used as sealing materials, particularly regarding changes in specific surface area and porosity. Three bentonites were analyzed: Ca-bentonite from Texas (STx-1b), Na-bentonite from Wyoming (SWy-3), and Ca-bentonite from Jelsovy Potok, Slovakia (BSvk). Treatment with ZnCl_2_ solution led to ion exchange and the formation of up to ~30% simonkolleite, accompanied by a concurrent decrease in montmorillonite content by 9–30%. A suite of analytical techniques, including X-ray diffraction (XRD), scanning electron microscopy (SEM), thermogravimetric analysis (TGA), X-ray fluorescence (XRF), and energy-dispersive X-ray spectroscopy (EDS), was employed to characterize these transformations. The contamination mechanism of ZnCl_2_ involves an ion exchange of Zn^2+^ within the montmorillonite structure, the partial degradation of specific montmorillonite phases, and the formation of a secondary phase, simonkolleite. These transformations caused a ~50% decrease in specific surface area and porosity as measured by the Brunauer–Emmett–Teller (BET) nitrogen adsorption and Barrett–Joyner–Halenda (BJH) methods. The findings raise concerns regarding the long-term performance of bentonite-based barriers. Further research should evaluate hydraulic conductivity, mechanical strength, and the design of modified bentonite materials with improved resistance to Zn-induced alterations.

## 1. Introduction

Bentonites are clays that belong to the smectite group of minerals, with the 2:1 layered montmorillonite (Mnt) being the main component; the general formula for bentonites is (Na, Ca)_0.4_(Al_4_-xMg_x_Si_8_-xFe_x_O_20_)(OH)_4_·nH_2_O. The structure of Mnt consists of two layers of silicon oxide (formed by tetrahedral units) with a layer of aluminum oxide (in an octahedral structure) in between. Substitution of atoms (x), such as aluminum (Al) with magnesium (Mg) or iron (Fe), creates a positive charge deficit, causing the Mnt layers to acquire a negative charge. Charge compensation mainly occurs through the adsorption of cations, such as sodium (Na) and calcium (Ca), onto the surface of the layers, which increases the size of the diffuse double layer (DDL) and enhances the mineral’s swelling capacity [1]. The unique sorptive properties and ability to exchange ions in bentonites make them valuable in various industrial fields, including medicine, construction, and materials technology, as well as having environmental applications [2,3,4,5]. One important use of Na-bentonites (>70% Mnt) is as a component of geosynthetic clay liners (GCLs), which are employed as sealing materials in landfills [6]. Egglofstein [7] observed that sodium ions in bentonite are replaced by Ca ions from cover soil through ion exchange over approximately 1–3 years. This results in a slight increase in the hydraulic conductivity of the bentonite barrier. Additionally, high-quality Na-bentonite, such as Wyoming bentonite (SWy-3), is widely used in the United States to produce barrier materials. However, it is not available on a large scale in other countries, where lower quality Ca-bentonites predominate [8]. These observations emphasize the importance of researching both Na- and Ca-bentonites used as GCLs. The widespread use of this material for sealing industrial waste landfills highlights the need to study the impact of potentially toxic metals (PTMs) and leachates with different chemical compositions on bentonite behavior [9,10].

Zinc(II) chloride (ZnCl_2_) is a common industrial waste generated as a by-product of textile waste recycling [11], as well as from galvanization processes and the dry battery industry [12,13]. As such, it can accumulate in landfills, where its presence poses a potential environmental risk. In industrial areas or closed mining sites, authors of different studies observed concentrations of Zn up to 5000 mg/kg [14], up to 7300 mg/kg [15], or up to 10,638 mg/kg [16] in various types of soils. Bentonites, due to their high specific surface area, exhibit increased reactivity, which can lead to the accumulation of significantly higher concentrations of PTMs, including Zn, particularly over extended exposure periods [8,13,17]. The mechanism of Zn adsorption includes both physical and chemical adsorption [18,19]. Physical adsorption involves the attraction of Zn ions to the surface of bentonite particles through van der Waals forces and ion exchange between Zn and metal cations in bentonite (mainly Na and Ca), which allows for their retention on the bentonite surface. Chemical adsorption, on the other hand, involves the formation of more stable Zn complexes, such as with oxygen groups (Si–O, Al–O) and hydroxyl groups (–OH). It is also possible for Zn complexes to form with Cl^−^ present in the environment, which has a limited tendency to bind with the solid phase of the soil [19]. Studies by Sun et al. [13] on bentonites contaminated with different concentrations of ZnCl_2_ (100–600 mg/L) showed that Zn adsorption is primarily the result of cation exchange regardless of the pH range (3–8). Chai et al. [20] observed that at pH ≥ 8, Zn adsorption is associated with the formation of metal complexes (such as Zn(OH)^+^, Zn(OH)_3_^−^, etc.) and the precipitation of an insoluble zinc hydroxide (Zn(OH)_2_). Similarly, Kaya and Ören [21] observed that between pH 4 and 7, the basic mechanism is an ion exchange process. At higher pH values (i.e., pH 8), it may lead to the formation of zinc hydroxyl species.

Huang et al. [3] indicated that an important direction for future research is assessing the impact of ion substitution in the structure of clay minerals on their behavior. Cui and Chen [8] suggested that high concentrations of Zn when exposed to a reactive bentonite barrier may alter its original properties. Kaya and Ören [21] observed that the negative charge on the clay surface is partially neutralized by positively charged Zn ions, which reduces the repulsion between clay particles and promotes the formation of aggregates. The research by Chai et al. [20] confirmed that 90% of bentonite particles (d_90_) have a diameter smaller than 2.79 μm. However, following the absorption of Zn ions, this value increases to 5.38 μm. X-ray diffraction analysis (XRD) conducted after the adsorption of 600 mg/dm^3^ Zn showed an increase in the basal spacing from 1.4471 nm to 1.48661 nm. These findings suggest that Zn ions were adsorbed into the interlayer due to electronic attraction. The study conducted by Nartowska et al. [17] confirmed an increase in the particle size of bentonite and a decrease in the clay fraction (CLY) after saturation with ZnCl_2_. Depending on the type of bentonite, d_10_ (the particle diameter below which 10% of the material’s mass is found) increases from 0.96 to 2.83 μm; CLY decreases from 42.6% to 6.6% for SWy-3 bentonite (Na-bentonite from Wyoming, USA), and d_10_ rises from 1.39 to 1.91 μm; CLY decreases from 18.5% to 10.7% for STx-1b bentonite (Ca-bentonite from Texas, USA), and d_10_ increases from 1.33 to 2.18 μm; and CLY decreases from 19.8% to 8.9% for BSvk bentonite (Ca-bentonite from Jelsovy Potok, Slovakia). Although the studies presented focus on different types of bentonites and vary in the concentrations of Zn achieved through different saturation methods, the results consistently indicate a concerning increase in the size of clay particles due to the bentonite’s Zn ion saturation. This may increase porosity, reduce the effectiveness of the clay geotechnical barrier, and pose an environmental threat. Further analysis is required to assess how ZnCl_2_ contamination, a common industrial contaminant, affects porosity and what the causes of this observed variability are.

In recent years, there has been growing interest in Zn-enriched bentonites, which may exhibit additional, previously unknown, properties, particularly in forming new mineral phases, such as simonkolleite (Zn_5_(OH)_8_Cl_2_·H_2_O) [22]. This mineral belongs to the layered double hydroxide salts, also known as “anionic clays.” It exhibits hydration and adsorption properties similar to natural clays, with a significant interlayer spacing (0.79 nm) [23]. Certain information regarding the presence of simonkolleite (Skl) has been reported in electronics and steel materials. Typically, Skl formation has been documented as a corrosion product of galvanized steel bolts exposed to a marine atmosphere [24]. However, Skl may form in systems where bentonite, zinc, and chloride ions coexist. For instance, Hsiao et al. [25] reported the formation of Skl in the zinc anode of a battery (Na-Mnt with a grain size below 10 μm) following treatment with hydrochloric acid. The formation of this mineral phase was associated with specific conditions. Oueslati et al. [26], in studies on the treatment of Wyoming bentonite (SWy-2) with a 0.5 M ZnCl_2_ solution, observed that a Skl signal appeared in XRD diffractograms. The authors conducted analyses on a single Na-bentonite sample and assessed its structural and hydration properties. The influence of Skl on the porosity and chemical properties of the bentonite was not considered.

The objective of this paper, explored for the first time, was to assess how ZnCl_2_-induced mineral phase formation influences the properties of bentonites used as sealing materials, particularly regarding changes in specific surface area and porosity. Understanding these effects is crucial for the design of sealing materials. While many studies have focused on the influence of Zn, pH, and granulometric composition on the quality of bentonite, there remains a critical need to assess porosity, which has not yet been evaluated in the case of bentonites containing Skl. Additionally, there is insufficient knowledge regarding the potential formation of these new mineral phases without additional treatments, such as temperature changes, HCl addition, particle size changes, or contact with steel corrosion products. This highlights the need for further research to document this mineral phase and assess its potential impact on bentonite quality, environmental safety, and the effectiveness of sealing materials. The novelty of this study lies in its unique focus on the in situ formation of Skl in ZnCl_2_-contaminated bentonites, without the use of additional treatments. This is the first study to explore the direct impact of this mineral phase on the structural and chemical properties of bentonites and its implications for geotechnical applications.

This study is based on the following research hypotheses:

1. Contamination of Na- and Ca-bentonites with ZnCl_2_ may lead to the formation of a new mineral phase—simonkolleite (Skl);
2. Saturation of bentonites with ZnCl_2_ leads to changes in their chemical composition, which may reduce their sorption capacity and the soil specific surface area (SSA);
3. The presence of Skl affects the porosity of the bentonites. This potentially alters their effectiveness in geotechnical barriers and poses a potential environmental threat.

## 2. Materials and Methods

### 2.1. Chemical Modification

The research material consisted of source clays obtained from the Clay Mineral Society (Purdue University, West Lafayette, Indiana), USA (Ca-bentonite from Texas (STx-1b) and Na-bentonite from Wyoming (SWy-3), as well as Ca-bentonite from Jelsovy Potok, Slovakia (BSvk)). All clays had particle sizes below 63 μm and contained at least 70% Mnt [17].

Fifty-gram samples were collected from each type of clay and subjected to an ion exchange process at room temperature (≈22 °C). Ten liters of a 1 mol L^−^¹ solution of ZnCl_2_ (Chempur, Poland, CAS No. 7646-85-7) (pH = 4.51 ± 0.01) was added to each sample, thoroughly mixed, and left to sit for 48 h. After this period, the clear liquid was decanted, leaving a sediment layer approximately 2 cm thick. Water was then carefully siphoned off from above the sediment, ensuring the sediment remained undisturbed. This procedure was repeated three times. The average pH value of the leachate from three decantations was 4.90 ± 0.07 for calcium bentonites and 5.05 ± 0.06 for sodium bentonite.

To eliminate chloride ions (Cl^−^) from the soil, the remaining material was placed in permeable membranes and stored in 20 L containers filled with circulating distilled water, allowing for Cl^−^ removal via osmosis. Cl^−^ levels were monitored using silver nitrate (AgNO_3_) until no Cl^−^ was detected. The water in the containers was changed every 2–3 days. The entire ion exchange process took approximately 35–40 days. Once the water no longer contained Cl^−^ ions, the soil pastes were transferred to glass cylinders and air-dried.

Additionally, the Cl^−^ concentration was analyzed in water extracts using the Mohr method. This method involves titration with silver nitrate (AgNO_3_) in the presence of chromate as an indicator, as described in PN-ISO 9297:1994 [27]. Before analysis, soil samples (2 g each) were shaken for 24 h on a rotary shaker in 20 mL of distilled water (conductivity 0.06 μS·cm^−1^). After shaking, the samples were filtered through Whatman filter paper, grade 1.6 μm, 50 mm diameter circle (Maidstone, Kent, UK), until a clear solution was obtained.

### 2.2. Mineralogical Analysis

The mineral composition of the tested clays was determined using X-ray diffraction (XRD) in the Bragg–Brentano geometry. X-ray diffraction patterns were recorded with a Bruker D8 Advance X-ray diffractometer (Bruker, Berlin, Germany) equipped with a Johansson-type monochromator (Bruker, Berlin, Germany) to generate CuKα1 radiation (λ = 1.5406 Å) and a position-sensitive LynxEye detector (Bruker, Berlin, Germany). Measurements were carried out in the 2θ range from 4.51° to 70° with 0.02° steps. The applied voltage was 3.540 kV, and the current was 530 mA. The identification of mineral phases in the clays was based on data from the PDF-4+ database of the International Center for Diffraction Data (ICDD, Newtown Square, PA, USA). The X-ray diffractograms of the clays were subjected to semi-quantitative mineralogical analysis using Diffrac Eva V6 software (Bruker, Berlin, Germany). Mineral content was calculated based on the intensity of their characteristic peaks in the diffractograms.

### 2.3. Thermal Analysis

Analysis of bentonites before and after ZnCl_2_ treatment was performed using a Thermal Gravimetric Analyzer SDT Q600 V20.9 (TA Instruments, New Castle, PA, USA). Before the measurements, calibrations were conducted to ensure the accuracy and reproducibility of the results: (1) DTA baseline correction, performed by the manufacturer’s protocol to minimize signal drift and ensure thermal stability across the temperature range (50–1200 °C) at a heating rate of 20 °C/min; (2) TGA weight calibration using certified standard weights; (3) temperature calibration using zinc as a reference material (melting point: 419.5 °C); and (4) cell constant adjustment to ensure optimal thermal response and signal consistency.

Samples of approximately 15 mg were placed in alumina pans. The reference sample was Calcium Oxalate Monohydrate (No. 900905.901, TA Instruments). The specimens were heated at a constant rate of 20 °C/min from 20 °C to 800 °C in a nitrogen atmosphere with a flow rate of 100 mL/min. The accuracy of the sample mass measurements was 0.1 μg, and the ∆T sensitivity (DTA) was 0.001 °C. Each bentonite sample was tested three times.

### 2.4. Scanning Electron Microscopy

The structural and chemical analysis was performed using scanning electron microscopy (SEM) with a field emission microscope JSM-7100F (JEOL, Tokyo, Japan) operating at a voltage of 15 kV. The surfaces of air-dried samples were coated with a thin layer of carbon to prevent charging using a JEOL JEC-530 sputter coater (JEOL, Tokyo, Japan). Images were analyzed at a magnification of ×5000. Such magnification allowed clear visualization of the morphology characteristic of simonkolleite, enabling its distinction from other phases present in the sample. To complement the analysis, SEM images were also acquired at magnifications of ×10,000 and ×30,000, allowing for a detailed assessment of the microcrystal morphology using the approach presented by Sun et al. [28].

### 2.5. Chemical Analyses of Bentonites

The chemical composition was analyzed using energy-dispersive X-ray spectroscopy (EDS) and X-ray fluorescence (XRF). inductively coupled plasma optical emission spectroscopy (ICP-OES) was also employed to support the interpretation of the results; the methodology and detailed procedure are comprehensively described in the authors’ previous publication [17]. The EDS method provides compositional information at the microscopic level, covering selected micro-areas of the sample. XRF was used to determine the elemental composition from the surface of the solid material. ICP-OES, in turn, enabled precise quantification of trace elements in the digested samples, thus complementing the data obtained by EDS and XRF.

In the case of EDS, the analysis was performed using an X-Max system (Oxford Instruments, Oxford, UK). Each element in the sample emits characteristic X-ray spectral lines when excited, with their energy distribution and intensity reflecting the type and concentration of the respective elements [29]. For the bentonite sample, a magnification of ×2000 was applied, corresponding to a scale of 50 µm visible in the SEM image. Under these conditions, the entire analyzed area, approximately 150 µm × 150 µm, was examined, enabling simultaneous assessment of the microstructure and the average chemical composition of several bentonite grains. An accelerating voltage of 20 kV was used, providing sufficient energy to excite characteristic X-ray emission from most elements typically found in bentonite (e.g., Si, Al, Mg, Fe, Ca, Na), thus allowing for a complete and representative analysis. This approach is particularly important for characterizing mineralogically heterogeneous materials.

The chemical composition of the clays was also analyzed using X-ray fluorescence (XRF) with a SPECTRO iQ II (Ametek, Meerbusch, Germany) equipped with a silicon drift detector (SDD) offering a resolution of 145 eV at 10,000 pulses. A Bragg crystal and Highly Ordered Pyrolytic Graphite (HOPG) target were used to polarize the primary beam. The measurements were conducted for 300 s under a helium atmosphere at voltages of 25 kV and 50 kV and currents of 0.5 mA and 1.0 mA, respectively. The analysis followed the standardized fundamental parameter method for powdered samples. The sample mass used for the analysis was approximately 2 g.

### 2.6. Granulometric and Plastic Properties

The physicochemical properties of clays are largely determined by the interactions between the solid phase (clay particles) and the liquid phase (water and dissolved substances) [17]. Their intensity depends on granulometric composition and plasticity parameters.

The granulometric composition was determined using a HELOS/BF SUCELL laser diffractometer from Sympatec GmbH (Clausthal-Zellerfeld, Germany), equipped with a “wet” test accessory. Clay samples were prepared as pastes 48 h before analysis to full saturation. For testing, 3 g of clay paste was mixed with 50 mL of distilled water, and a small sample was taken to ensure that the particle concentration in the test chamber did not exceed 25%. Reference measurements were performed before each new analysis. The effective diameter d_10_ was derived from the granulometric curves, indicating that 10% of the mass consisted of grains equal to or smaller than this diameter within the soil sample. This parameter is used to assess the filtration properties of soil, and smaller values generally indicate lower permeability.

The plasticity characteristics were measured using standard methods: Casagrande’s cup device for determining the liquid limit (LL) and the test for the rolling plastic limit (PL) according to standard no. EN ISO/TS 17892-12 [30].

### 2.7. Specific Surface Area and Porosity

The specific surface area of the clays (SSA, [m^2^/g]) studied was determined using the following:

- The water vapor sorption test (WST) according to Stępkowska [31] (SSA_WST_) (1),

(1) $${SSA}_{WST}=6\cdot (w_{50}\cdot 5.85)$$

where w_50_ is the sorption moisture at a vapor pressure $\frac{p}{p_{0}}$ = 0.5 in a desiccator over saturated magnesium nitrate (V) solution determined by drying at 220 °C (sorption takes 10 days).

Additionally, the sorptive moisture at $\frac{p}{p_{0}}$ = 0.95 was determined in a desiccator over a 10% sulfuric acid (VI) solution (with sorption taking 14 days). This moisture can be identified as strongly bound water.

- The Brunauer–Emmett–Teller (BET) method (SSA_BET_) (2)

(2) $${SSA}_{BET}=a_{m}\cdot \omega \cdot N_{A}$$

where a_m_ is the amount of adsorbed gas (N_2_), ω is the surface occupied by a single adsorbate molecule in a monomolecular layer, also known as the “molecular cross-sectional area”, and N_A_ is Avogadro’s number.

Additionally, the Barrett–Joyner–Halenda (BJH) model was used to determine the surface area of mesopores (SA_BJH_) using NOVA Touch Software firmware v1.09, S/N 1050020598 (Anton Paar GmbH, Graz, Austria). This model assumes that pores are filled with gas according to the principles of capillary condensation, which occurs as pressure increases. Based on the Kelvin equation, which relates vapor pressure to the curvature radius of the liquid meniscus, the pore size can be determined (3).

(3) $$\ln\left(\frac{p}{p_{0}}\right)=-\frac{2\gamma Vm}{rRT}$$

where γ is the surface tension of the liquid nitrogen, Vm is the molar volume, R is the gas constant, T is the temperature, and $\frac{p}{p_{0}}$ is the relative pressure.

The thickness of the adsorbate layer on the pore walls is then added to the capillary radius, assuming cylindrical pores. The analysis is performed using the nitrogen desorption isotherm, enabling the determination of the pore size distribution and pore volume. The mesoporous surface area is calculated as the sum of the surface areas of pores of specific sizes derived from this distribution.

The BET method was used to calculate the external specific surface area of the bentonites (SSA_BET_), which corresponds to the upper boundary of micropores (>0.7 nm) and includes mesopores (2–50 nm), where inter-packet spaces are not accessible to water. The WST test, on the other hand, provides more comprehensive information, accounting for both external and internal surfaces. The BJH method was applied to determine the surface area of mesopores (SA_BJH_). This parameter is important because it reflects the portion of the material’s surface potentially involved in ion exchange, adsorption, or catalytic processes, features not fully captured by BET when microporosity dominates. The accurate determination of SA_BJH_ helps distinguish surface area contributions from different pore size distributions, complementing the BET and WST results. The relevance of combined surface analyses (SA_BJH_ and SSA_BET_) was demonstrated in the studies by Asabina et al. [32] and Borinelli et al. [33].

SSA_BET_, SA_BJH_, and the pore space distribution of soil samples were investigated using low-temperature nitrogen (77 K; p/p0 = 0.047–0.95) adsorption–desorption, performed with a NOVA Touch 2LX (Anton Paar, Graz, Austria) instrument. Before measurements, samples weighing approximately 0.35 g were heated to 105.0 °C at a rate of 10.0 °C/min and then held at this temperature for 300 min for degassing.

### 2.8. Data Quality Control

Bentonites had grain sizes of d < 63 μm, ensuring high homogeneity and uniformity. Additionally, this study was conducted on two source clays, STx-1b and SWy-3, obtained from the Clay Mineral Society collection, guaranteeing their well-verified quality, stability, and representativeness as well-characterized reference materials. High-purity reagents (analytical or chemically pure, Chempur, Piekary Śląskie, Poland)), free from contaminants that could affect analytical results, were used throughout the analyses. Materials with known composition and structural properties provided a solid reference for monitoring structural transformations in bentonites contaminated with ZnCl_2_ (via XRD and TG analyses). Thermogravimetric analysis (TG) results were compared with X-ray diffraction (XRD) data, allowing a comprehensive interpretation of mineralogical changes and confirming the phases present in the studied samples. Chemical analyses were performed using ICP-OES and XRF techniques. For ICP-OES, a multi-element calibration standard (Instrument Calibration Standard 2, No. N9301721, PerkinElmer, Waltham, MA, USA) was employed, and calibration curves were evaluated using linear regression at a significance level of *p* = 0.01; Grubbs’s test was applied to exclude outliers. Three replicate measurements were performed for each bentonite specimen, and the calculated standard deviations (SD) were used as practical estimates of measurement uncertainty. Similarly, XRF measurements were conducted in triplicate. All resulting standard deviations are reported in Section 3.2. In SEM-EDS analyses, ZAF correction and normalization were applied to compensate for matrix effects and reduce systematic errors. Elemental composition was determined over the largest possible representative area of the sample, allowing averaging of results and minimizing the influence of local heterogeneities. For specific surface area measurements by the BET method, uncertainty was indirectly estimated based on the quality of the BET model fit by analyzing parameters such as slope, intercept, and the BET constant (C). A high correlation coefficient (r = 0.99) indicates excellent agreement of the model with the data, confirming the reliability and accuracy of the obtained surface area values.

## 3. Results and Discussion

### 3.1. Mineralogical and Microstructural Analysis

#### 3.1.1. XRD

**Figure 1:**
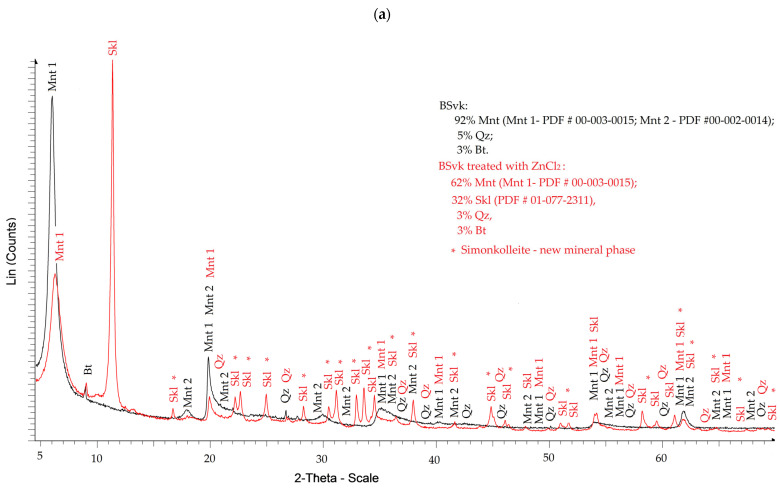

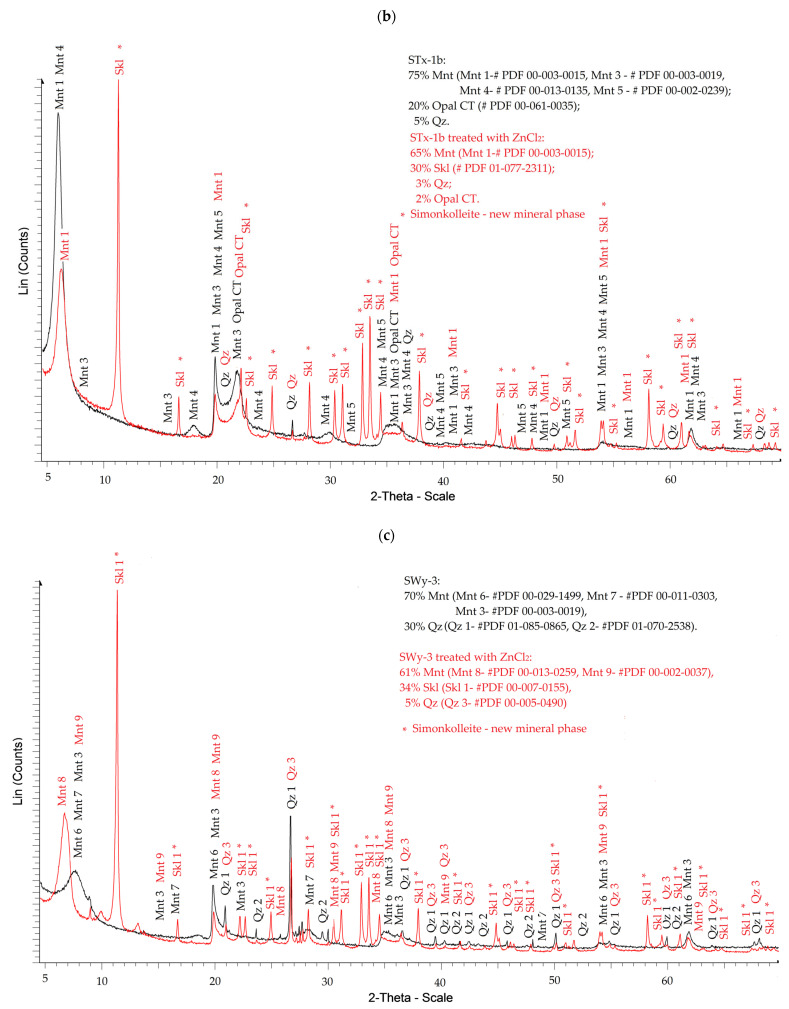
XRD diffractograms of bentonites before and after treatments with ZnCl_2_: (**a**) natural calcium bentonite from Jelsový Potok, Slovakia (BSvk), (**b**) natural calcium bentonite from Texas, USA (STx-1b), (**c**) natural sodium–calcium bentonite from Wyoming, USA (SWy-3).

Figure 1 shows the XRD diffractograms for bentonites before and after contamination with ZnCl_2_. XRD-identified mineral phases in bentonites, along with corresponding PDF references, are listed in Table A1. The montmorillonite content (Mnt) in uncontaminated SWy-3 and STx-1b is nearly consistent with the values reported by Chipera and Bish [34] (SWy-2—75% Mnt; STx-1—67% Mnt) and by Castellini et al. [35] (STx-1b—73% Mnt).

In bentonites contaminated with ZnCl_2_, a new mineral phase, Skl, forms and coexists in an amount of approximately 30% with the main mineral, Mnt (Figure 1a–c). Simonkolleite occurring in calcium (Ca) bentonites (PDF #01-077-2311) and sodium (Na) bentonites (PDF #00-007-0155) exhibits minor differences in crystallographic parameters, which may influence the physical properties of these bentonites. Considering the general formula of Mnt, the probable mechanism for the formation of Skl from Mnt can be predicted:

1. Saturating montmorillonite (pH 7.92 ± 0.12) with a ZnCl_2_ solution (pH = 4.51 ± 0.01) causes only a slight increase in the suspension’s pH to 4.98 ± 0.11. Under these mildly acidic conditions, intensive ion exchange and partial dissolution of montmorillonite components occur. Chemical reactions, such as the formation of Zn(OH)_2_ according to Equation (4), take place primarily at the montmorillonite phase boundary or within its structure, where the local pH is higher.

(4) $$Zn{Cl}_{2}+2OH^{-}\to Zn{\left(OH\right)}_{2}+{2Cl}^{-}$$

2. The formation of Skl is most likely caused by Zn ions, which partially replace Al, Mg, Fe, and Si ions (as indicated by the XRF data described in the next section) in the Mnt structure, as well as SiO_2_, as shown by the XRD data (Figure 1). Only a portion of Mnt and SiO_2_ contributes to the formation of Skl, so the general reaction equation may proceed according to scheme (5).

(5) $$Zn{\left(OH\right)}_{2}+{2{Cl}^{-}+Mnt}^{*}+SiO_{2}\to {Skl}^{**}+the\;remaining\;Mnt+the\;remaining\;SiO_{2}$$

Notes: The general formula for Mnt montmorillonite is * (Na, Ca)_0.4_(Al_4−_xMg_x_Si_8−_xFe_x_O_20_)(OH)_4_·nH_2_O; the general formula for Skl simonkolleite is ** (Zn_5_(OH)_8_Cl_2_·H_2_O).

A decrease in silica content is also observed, likely associated with its dissolution. Bagheri et al. [36] showed that at alkaline pH values, calcium increases the rate of silica dissolution, while aluminum slows it down. Since the greatest decrease in silica content was observed in the Texas bentonite (Figure 1b), it can be assumed that one possible reason is the presence of the highest concentration of calcium ions and the lowest concentration of aluminum ions in this sample (Section 3.2) [17].

To evaluate the behavior of the Mnt phases as a result of their saturation with ZnCl_2_, Table A1 was analyzed, which summarizes their occurrence.

As shown in Figure 1a, the Mnt 2 montmorillonite phase (NaMgAlSiO_2_(OH)H_2_O, PDF no. 00-002-0014) disappears from the XRD pattern in the BSvk sample after the treatment with ZnCl_2_. This phenomenon is likely due to the destabilization of the Mnt 2 structure caused by the exchange of Na and Mg cations for Zn. The Zn ion has a higher charge and a different ionic radius compared to Na and Mg—its ionic radius (~0.74 Å) is slightly larger than that of Mg (~0.72 Å) but significantly smaller than that of Na^+^ (~1.02 Å). This results in stronger electrostatic interactions between the mineral layers and introduces structural strain, disrupting the regular arrangement of the montmorillonite layers. Consequently, the interlayer spacing contracts, and the crystalline order is lost, leading to the disappearance of characteristic peaks in the XRD pattern. On the other hand, the Mnt 1 phase with the composition (Na, Ca)_0.3_(Al, Mg)_2_Si_4_O_10_(OH)_2_·xH_2_O (PDF no. 00-003-0015) shows greater stability. The presence of Ca (~1.00 Å) and the mixed cation composition provide increased structural resistance to cation exchange and disturbances. Ca, with its larger ionic radius and charge, stabilizes the layer structure, maintaining the regular arrangement of layers despite the presence of Zn. As a result, this phase retains its crystallinity and its peaks remain visible in the XRD analysis.

Selected forms of montmorillonite—Mnt 3: Na-Al-Si-O-OH-H_2_O (PDF no. 00-003-0019); Mnt 4: 15 Å Ca_0.2_(Al, Mg)_2_Si_4_O_10_(OH)_2_·4H_2_O (PDF no. 00-013-0135); and Mnt 5: CaMg_2_AlSi_4_(OH)_2_·H_2_O (PDF no. 00-002-0239)—likely undergo crystal structure breakdown or transform into an amorphous state as a result of the reaction of STx-1b with ZnCl_2_ (Figure 1b). This process is driven by intense ion exchange, where Zn displaces Na, Ca, and Mg from the interlayer spaces. Removing interlayer cations without effective compensation of the negative charge in the tetrahedral sheets leads to the weakening of interlayer bonds and the gradual disintegration of the crystal structure. These changes result in the loss of layered ordering, observed in XRD analysis as a decrease in intensity or complete disappearance of diffraction peaks characteristic of Mnt 3, 4, and 5 (Figure 1b). In contrast, the Mnt 1 phase ((Na, Ca)_0.3_(Al, Mg)_2_Si_4_O_10_(OH)_2_·xH_2_O, PDF no. 00-003-0015) showed no significant changes in its crystal structure after treatment with ZnCl_2_—only a reduction in peak intensity was noted, which may indicate a decrease in crystalline material quantity or partial dehydration due to Zn incorporation. This suggests that secondary mineralization of simonkolleite (Zn_5_(OH)_8_Cl_2_·H_2_O) occurs independently of this particular montmorillonite form and does not significantly affect its structural stability.

Based on Figure 1c, in the SWy-3, the Mnt 6—22 Å montmorillonite phase (Na_0.3_(Al, Mg)_2_Si_4_O_10_(OH)_2_·H_2_O, PDF no. 00-029-1499) undergoes structural modifications as a result of intensive ion exchange triggered by the introduction of ZnCl_2_ into the bentonite matrix. This process is accompanied by a decrease in pH (from 8.05 to 6.84) and an increase in salinity, which further facilitates the Mnt phase transformation. As a result, a transition occurs to a new, structurally equivalent Mnt 8—14 Å montmorillonite phase (Na_0.3_(Al, Mg)_2_Si_4_O_10_(OH)_2_·xH_2_O, PDF no. 00-013-0259)—characterized by reduced interlayer water content and altered physicochemical properties. Additionally, other montmorillonite-related phases identified in the SWy-3, Mnt 7—(Al(OH)_2_)_0.33_Al_2_(Si_3_.₆₇O_10_)·8H_2_O (PDF no. 00-011-0303)—and Mnt 3—Na-Al-Si-O-OH-H_2_O (PDF no. 00-003-0019)—are likely transformed or degraded into a deionized or structurally simplified aluminosilicate phase such as Mnt 9—AlSi_2_O₆(OH)_2_ (PDF no. 00-002-0037)—due to intensive ion exchange and partial dehydration. The release of OH^−^ ions and structural degradation products from the aluminosilicate framework promotes the crystallization of Skl (Zn_5_(OH)_8_Cl_2_·H_2_O) as a secondary mineral phase, likely forming as microscale aggregates within the pores and interlayer spaces of montmorillonite. These structural transformations are also reflected in changes in the interlayer spacing (d_001_), as summarized below.

The highest interlayer distances of Mnt (d_001_) before saturation with ZnCl_2_ were 14.87, 14.88, and 11.44 Å, while after saturation, they measured 14.29, 14.17, and 13.21 Å for the bentonites BSvk, STx-1b, and SWy-3, respectively [17]. After saturation, a slight decrease in interlayer spacing was observed in Ca-Mnt, which may be due to the smaller ionic radius of Zn compared to Ca. Voora et al. [37] showed that the larger the interlayer cation radius (Li, Na, K, Mg, Ca), the greater the interlayer distance in Mnt. However, our study did not confirm this relationship for Na-Mnt, where, despite the smaller ionic radius of Zn (0.74 Å) compared to Na (0.95 Å), an increase in interlayer spacing was observed. This may be due to microstructural changes and improved hydration of Zn ions in Na-bentonite after saturation with ZnCl_2_, leading to an increase in the effective ionic radius. Similarly, Oueslati et al. [26] observed larger interlayer distances in ZnCl_2_ contaminated SWy-2 bentonite with rises in relative humidity (15–75%). The results may also suggest that in SWy-3, Zn ions mainly adsorb in the interlayer spaces of Mnt, most likely due to the easier penetration of Zn ions, which have a smaller ionic radius than Na, potentially leading to an increase in interlayer distance. The main mechanisms responsible for this process may include electronic attraction and interlayer cation exchange [20]. In contrast, in STx-1b and BSvk, Zn primarily adsorbs on the surface of Mnt particles because in Ca-bentonites, the larger radius of Ca (compared to Na) makes it more difficult for Zn ions to access the interlayer spaces. However, this does not exclude the involvement of Zn in cation exchange in these Mnt types, as indicated by the observed, albeit slight, decrease in interlayer spacing, the high Zn content in the saturated samples, and its presence across the entire surface shown in the EDX images, which will be presented in the following section of this work (Section 3.2).

#### 3.1.2. TGA

**Figure 2:**
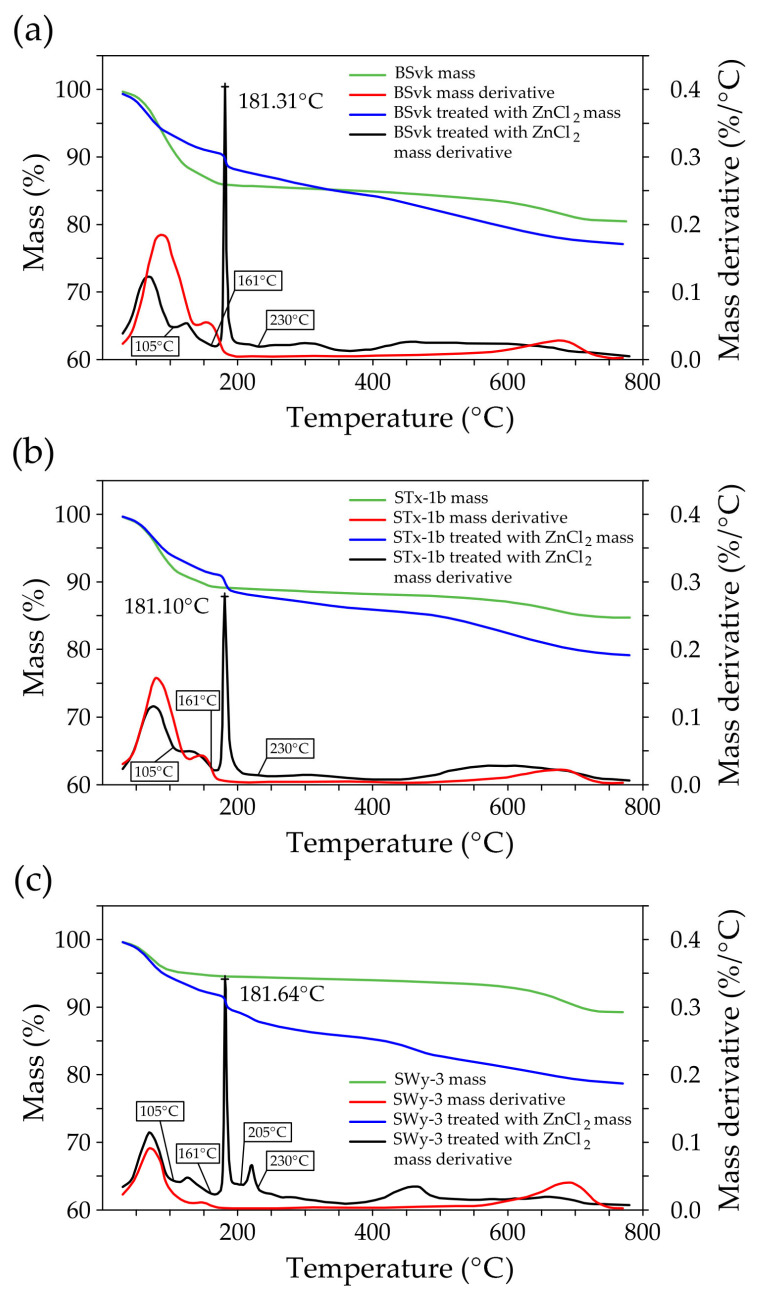
TG and DTG curves of bentonite before and after treatments with ZnCl_2_: (**a**) natural calcium bentonite from Jelsový Potok, Slovakia (BSvk), (**b**) natural calcium bentonite from Texas, USA (STx-1b), (**c**) natural sodium–calcium bentonite from Wyoming, USA (SWy-3).

The thermogravimetry (TG) and derivative thermogravimetry (DTG) curves of bentonites, both before and after treatment with ZnCl_2_, are shown in Figure 2. For Mnt, three thermal decomposition steps were recorded in bentonites (Figure 2):

1. Desorption (at ~75 °C): This stage corresponds to the release of physically adsorbed water (molecular water on or within clay mineral particles) due to weak interactions, such as van der Waals forces or hydrogen bonds.
2. Dehydration (at ~150 °C): In this step, water that was desorbed from the mineral surface, potentially weakly bound by electrostatic forces or other physical interactions, is further released. This water may include interlayer water between the mineral layers, although it is not chemically bonded to the Mnt. In Zn-bentonites, the dehydration peak occurs at around 125 °C, which may indicate structural changes in the Mnt layers due to the interaction with Zn ions.
3. Thermal decomposition between 550 °C and 800 °C can be attributed to dehydroxylation which refers to the release of chemically bound water associated with the mineral’s structure. This water is part of the hydroxyl groups (-OH) in the Mnt layers, forming part of the interlayer cations and the mineral structure.

**Figure A1:**
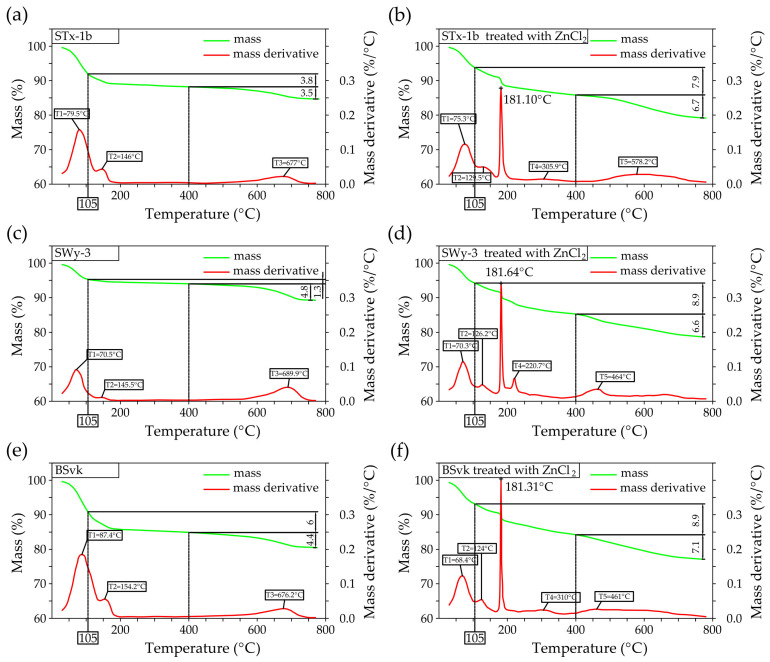
TG and DTG curves of bentonite before and after treatments with ZnCl_2_: (**a**) natural calcium bentonite from Texas, USA (STx1b), before and (**b**) after; (**c**) natural sodium–calcium bentonite from Wyoming, USA (SWy-3), before and (**d**) after; (**e**) natural calcium bentonite from Jelsový Potok, Slovakia (BSvk), before and (**f**) after.

The maximum peak temperatures for each step of the thermal decomposition of Mnt depend on the type of bentonite and are as follows for BSvk, STx-1b, and SWy-3: (desorption) T_1_ = 87.4 °C, 79.5 °C, 70.5 °C; (dehydration) T_2_ = 154.2 °C, 146 °C, 145.5 °C; (dehydroxylation) T_3_ = 676.2 °C, 677 °C, and 689.9 °C. Contamination of the bentonites with ZnCl_2_ lowered the dehydration temperature of Mnt (T_2_ = 124 °C, 129.5 °C, 126.2 °C) (Figure 2, Figure A1). This phenomenon can be explained by the osmotic forces created after the contamination was introduced into the bentonite and by structural changes. Higher ionic strength weakens the electrostatic repulsion between particles, allowing for easier migration of molecules and ions through the material, which may contribute to the lowering of the dehydration temperature. A similar phenomenon was observed by Zhu et al. [38], who studied bentonite mixtures contaminated with Cr(VI) and found that the compression of the diffusion double layer (DDL) under high ionic strength conditions enlarges the diffusion path, enhancing the migration of Cr(VI) through the pore flow paths.

As shown in Figure 2a–c, ZnCl_2_ contamination alters the shape of the dehydroxylation peak. In the bentonites BSvk, STx-1b, and SWy-3, dehydroxylation occurred at temperatures above 550 °C prior to contamination. After treatment, the SWy-3 sample exhibits a distinct peak in the range of 400–500 °C, which can be attributed to the thermal instability of the secondary phase—simonkolleite (T > 400 °C [23])—along with a broader montmorillonite dehydroxylation peak beginning at around 480 °C. In contrast, in the BSvk and STx-1b samples, a broad dehydroxylation peak is observed across a wide temperature range above 400 °C. This is most likely the result of the overlap between the simonkolleite decomposition peak and the montmorillonite dehydroxylation peak, the precise onset of which is difficult to determine. However, the shape of the peaks indicates a lowering of the montmorillonite dehydroxylation temperature in all the studied bentonites. This phenomenon can be attributed to two overlapping effects: (1) direct interactions of Zn^2+^ ions with the aluminosilicate layers, which disrupt interlayer bonding and weaken the mineral’s structural framework, an effect enhanced by the strong hydration shell of Zn [39], and (2) microstructural changes caused by the decomposition of simonkolleite, leading to the formation of defects and local distortions in the vicinity of montmorillonite. Both factors reduce the energy required to initiate dehydroxylation, resulting in its occurrence at lower temperatures.

Thermogravimetric measurements confirm the formation of Skl in ZnCl_2_-treated bentonites (Figure 2). At temperatures ranging from 105 to 161 °C, Skl loses part of its water (H_2_O) and transforms into the form Zn_5_(OH)_8_Cl_2_ (6).

Simonkolleite (Skl)

(6) $${Zn}_{5}{\left(OH\right)}_{8}{Cl}_{2}\cdot H_{2}O\overset{105-161{}^{\circ}C}{\to }{Zn}_{5}{\left(OH\right)}_{8}{Cl}_{2}+H_{2}O$$

A peak in the temperature range from 161 to 200 °C (with a maximum of 181 °C) corresponds to the first decomposition of Skl (7).

(7) $${Zn}_{5}{\left(OH\right)}_{8}{Cl}_{2}\overset{161-200{}^{\circ}C}{\to }{3ZnO+ZnO\cdot ZnCl}_{2}\cdot 2H_{2}O+{2H}_{2}O$$

Analogous to the mechanism proposed by Moezzi et al. [23] for Skl, it is suggested that for bentonite containing Skl (Zn SWy-3—Na-bentonite treated with ZnCl_2_), in the temperature range of 205–230 °C, part of the water is released from the mineral ZnO·ZnCl_2_·2H_2_O. As a result, the mineral structure transitions to the form Zn(OH)_2_·ZnCl_2_·2H_2_O, which is observed as a distinct peak on the graph (e.g., in Figure 2c) (8).

(8) $$ZnO\cdot Zn{Cl}_{2}{\cdot 2H}_{2}O\overset{205-230{}^{\circ}C}{\to }{Zn{\left(OH\right)}_{2}\cdot ZnCl}_{2}\cdot 2H_{2}O+H_{2}O$$

The subsequent gradual mass loss observed in the treated SWy-3 above 400 °C can be attributed to the decomposition of a new amorphous phase (9).

(9) $$Zn{\left(OH\right)}_{2}\cdot Zn{Cl}_{2}\left(amorphous\right)\overset{T>400{}^{\circ}C}{\to }2ZnO+2HCl$$

In Zn STx-1b and Zn BSvk (Ca-bentonites treated with ZnCl_2_), the peak in the 205–230 °C temperature range is not as distinct as in Zn SWy-3, which may suggest that in these clays, water removal occurs gradually. The larger size of the Ca ion (1.12 Å) compared to Na (0.95 Å) leads to stronger bonds with the bentonite layers, making the exchange of Zn ions more difficult. Zn ions (0.74 Å) that have been exchanged in Ca-containing bentonite become more stable as a result, and the bentonite structure is more difficult to change. This may also contribute to the difficulty in releasing water and Zn ions [17].

Since in ZnCl_2_-treated bentonites dehydration occurs at temperatures above 105 °C and dehydroxylation occurs above 400 °C, mass loss in these temperature ranges was analyzed to assess differences between untreated and ZnCl_2_-treated bentonites and to demonstrate the presence of Skl (Figure A1). In the 105–400 °C range, mass losses were observed for BSvk, STx-1b, and SWy-3 before (6.0%, 3.8%, 1.3%) and after (8.9%, 7.9%, 8.9%) ZnCl_2_ contamination. In the 400–800 °C range, similar increases were observed: before (4.4%, 3.5%, 4.8%) and after (7.1%, 6.7%, 6.6%) treatment. These higher mass losses indicate the formation of simonkolleite. The newly formed mineral phase exhibits thermal properties distinct from montmorillonite, as evidenced by differences in thermal decomposition. Montmorillonite typically loses thermal stability at temperatures above 550 °C. In contrast, secondary phases such as simonkolleite decompose at significantly lower temperatures, starting at 161 °C, with complete decomposition occurring above 400 °C. This process releases Zn, which may affect the ability of ZnCl_2_-treated bentonite to retain zinc under these conditions. We cannot exclude the fact that these thermal processes also induce changes in the microstructure, chemical composition, and physical properties of bentonite, further impacting the stability of the matrix.

The total mass loss after the decomposition of Skl in the studied bentonites ranges from 21.7% to 23.3%. Previous studies report values between 25.5% and 37.5%, but these refer only to the Skl phase. The authors explain that the mass loss depends on how chlorine behaves during the decomposition process. If chlorine is released as HCl, the mass loss is smaller, whereas if chlorine transforms into ZnCl_2_ and then evaporates, the mass loss is greater [23].

#### 3.1.3. SEM

**Figure 3:**
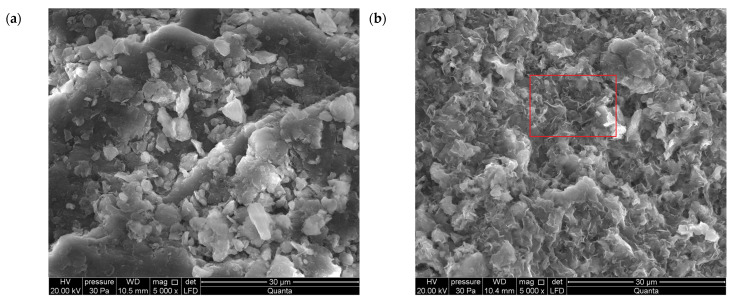

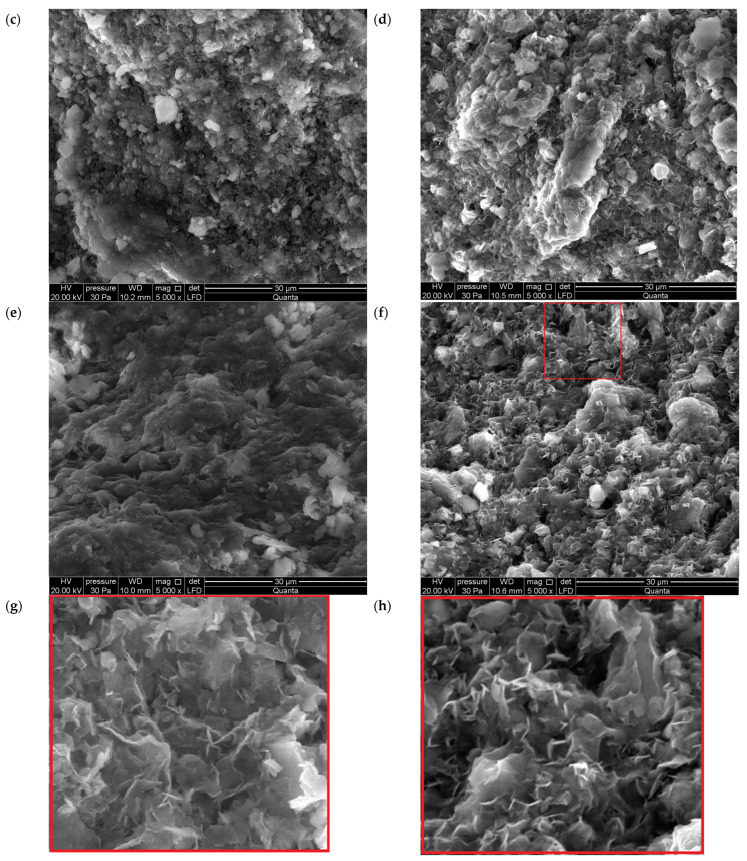
SEM microphotographs of bentonites before and after treatment with ZnCl_2_: (**a**) calcium bentonite from Jelsový Potok, Slovakia (BSvk), (**b**) BSvk after treatment, (**c**) calcium bentonite from Texas, USA (STx-1b), (**d**) STx-1b after treatment, (**e**) sodium bentonite from Wyoming, USA (SWy-3), (**f**) SWy-3 after treatment, (**g**) the needle-like structure of simonkolleite in BSvk bentonite after treatment, (**h**) the needle-like structure of simonkolleite in SWy-3 bentonite after treatment.

In the SEM images (Figure 3b,d,f–h) of bentonites treated with ZnCl_2_, thin-layered plates are visible that correspond to the needle-like structure of Skl [40]. Aggregates with a noticeable degree of ordering can be observed within the structure. The micrographs of Zn-saturated bentonites exhibit better electron-reflecting ability compared to the unsaturated bentonites. In the latter (Figure 3a,c,e), schistose structures, chaotically arranged aggregates, and low contrast between the layers are observed. The formation of aggregates in Zn-contaminated bentonites was also documented by Chai et al. [20]. The process of clay particle aggregation can be explained by the adsorption of Zn ions onto the clay surface, which neutralizes part of its negative charge, reducing repulsion between particles.

**Figure 4:**
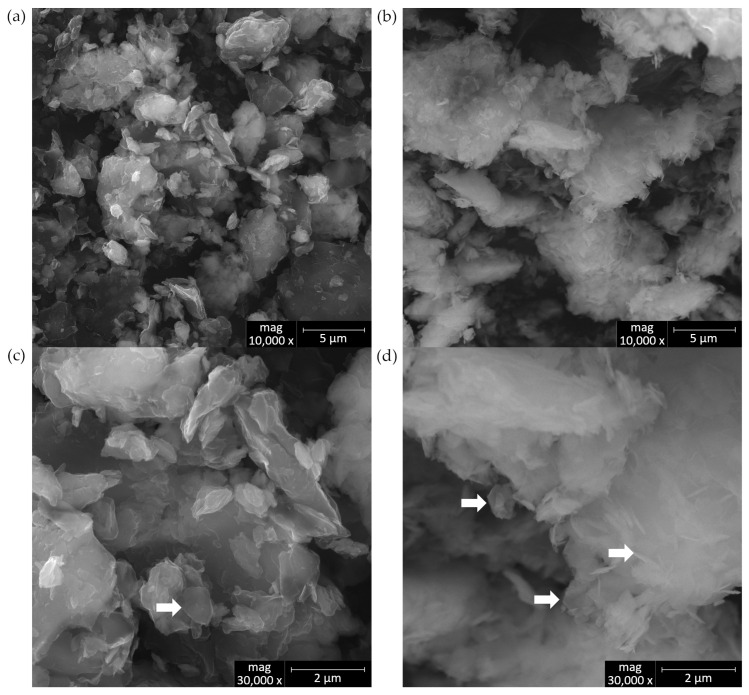
Microcrystal morphology of simonkolleite (Skl) observed in SEM microphotographs of bentonites after treatment with ZnCl_2_: (**a**,**c**) sodium bentonite from Wyoming, USA (SWy-3); (**b**,**d**) calcium bentonite from Jelsový Potok, Slovakia (BSvk). Note: The white arrow indicates hexagonal platelets of simonkolleite.

Simonkolleite is a mineral that naturally occurs in thin flakes, scales, or layers, forming crystalline aggregates ranging in size from several tens of nanometers to a few micrometers. These aggregates most commonly adopt a regular hexagonal platelet morphology; however, in practice, more complex structures may also arise due to the crystal growth process. Figure 4 illustrates the morphology of the obtained aggregates at higher magnifications (scale bars: 5 µm and 2 µm), allowing for a detailed analysis of their structure and uniformity. It should be noted, however, that most current synthesis methods for simonkolleite require precise pH adjustment of zinc salt solutions in an alkaline environment. Despite these efforts, such methods often result in not only the desired hexagonal morphologies but also undesired forms with irregular shapes, making it difficult to control the final structure of the material [41].

### 3.2. Chemical Analysis

**Figure 5:**
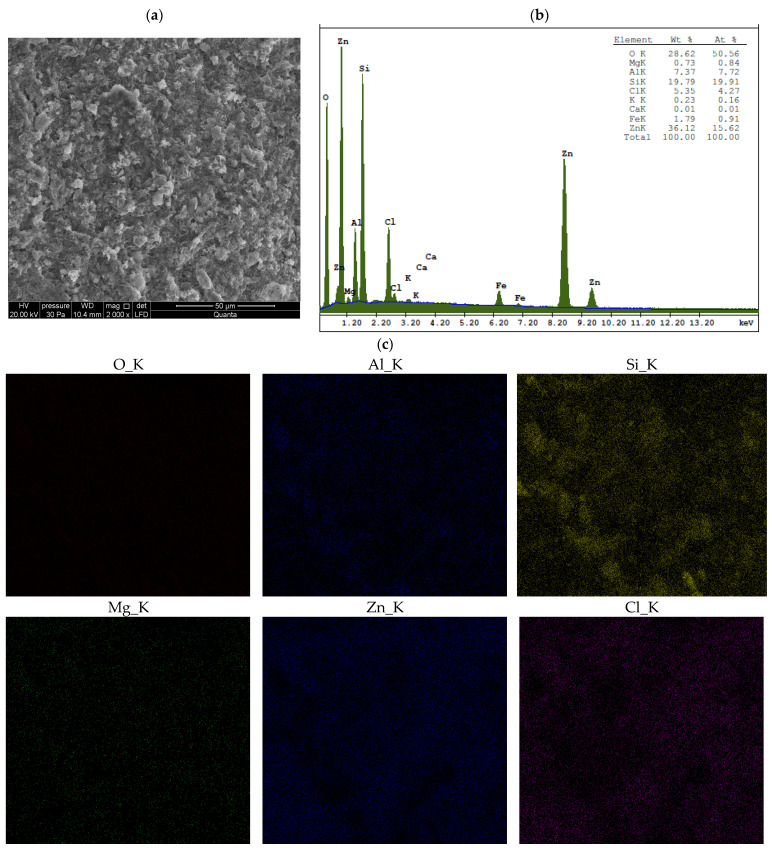
EDS analysis of BSvk bentonite after treatment with ZnCl_2_: (**a**) area of analysis with microphotograph; (**b**) elemental composition spectrum; (**c**) spatial distribution of selected elements on the sample surface.

**Figure 6:**
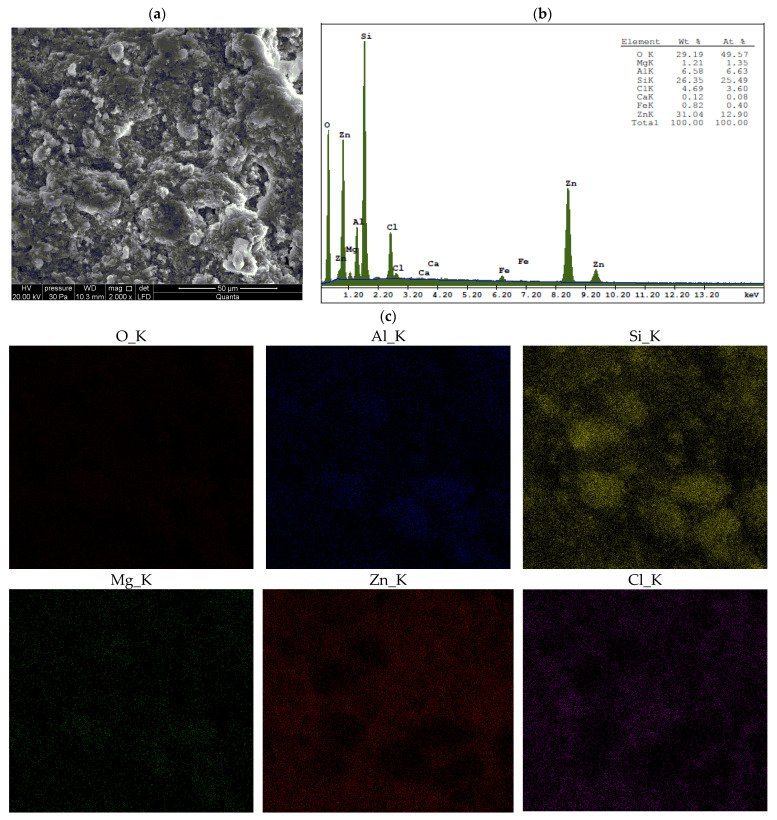
EDS analysis of STx-1b bentonite after treatment with ZnCl_2_: (**a**) area of analysis with microphotograph; (**b**) elemental composition spectrum; (**c**) spatial distribution of selected elements on the sample surface.

**Figure 7:**
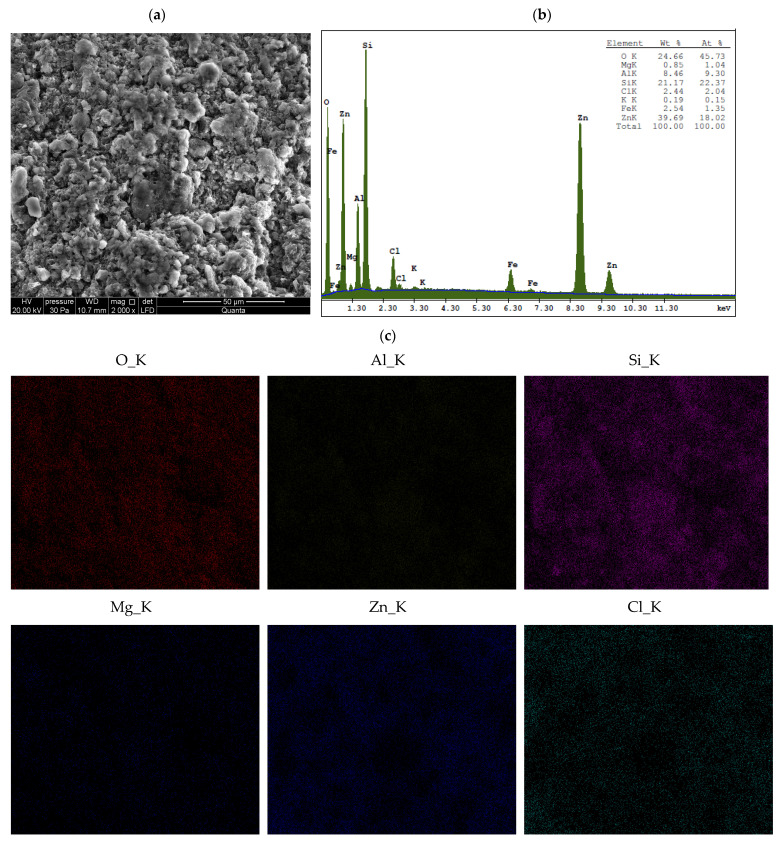
EDS analysis of SWy-3 bentonite after treatment with ZnCl_2_: (**a**) area of analysis with microphotograph; (**b**) elemental composition spectrum; (**c**) spatial distribution of selected elements on the sample surface.

**Figure A2:**
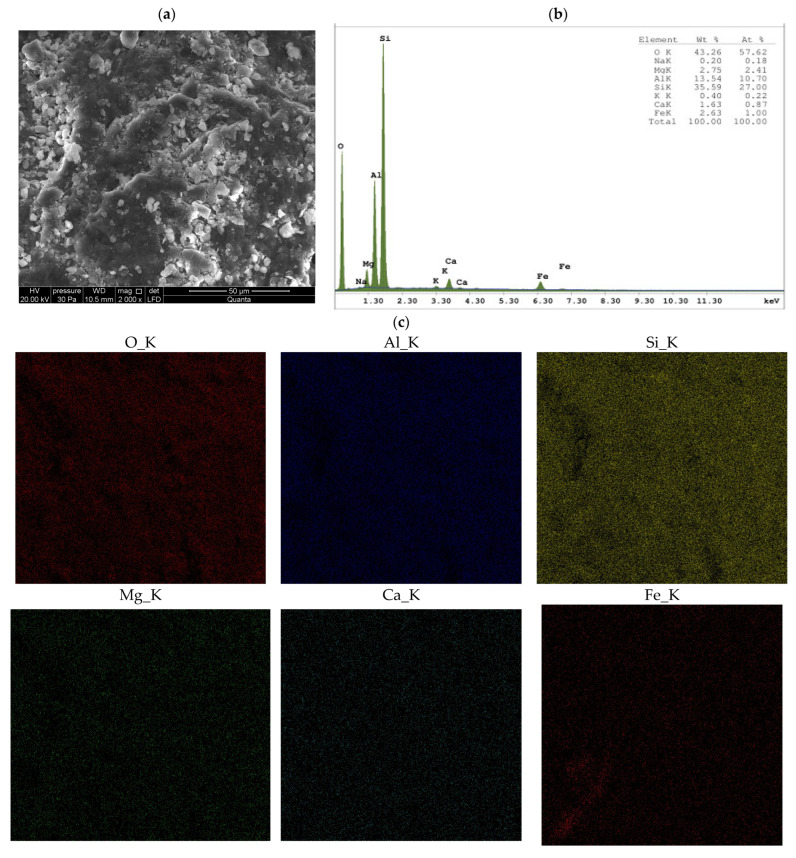
EDS Analysis of BSvk bentonite before treatment with ZnCl_2_: (**a**) area of analysis with microphotograph; (**b**) elemental composition spectrum; (**c**) spatial distribution of selected elements on the sample surface.

**Figure A3:**
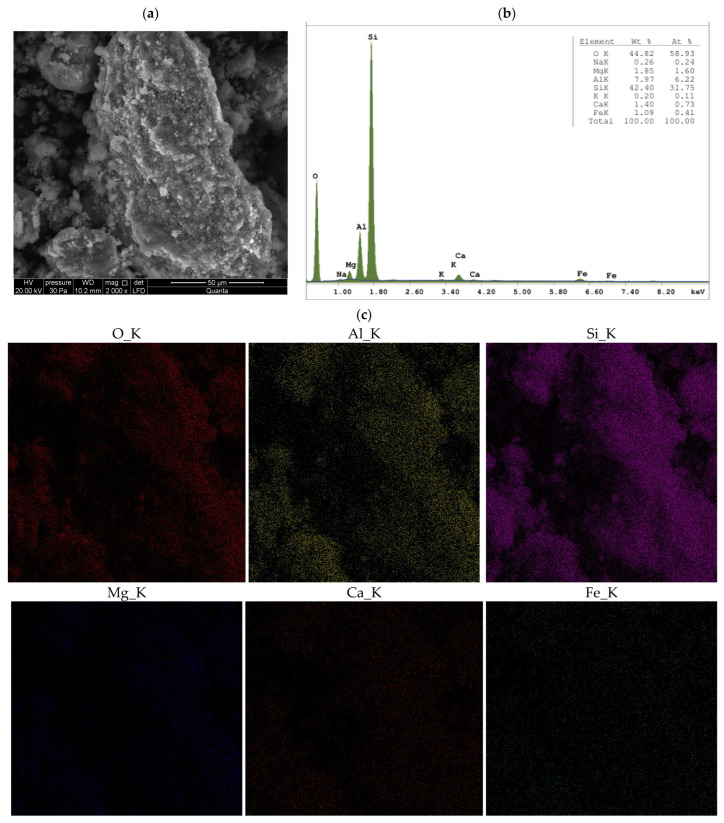
EDS Analysis of STx-1b bentonite before treatment with ZnCl_2_: (**a**) area of analysis with microphotograph; (**b**) elemental composition spectrum; (**c**) spatial distribution of selected elements on the sample surface.

**Figure A4:**
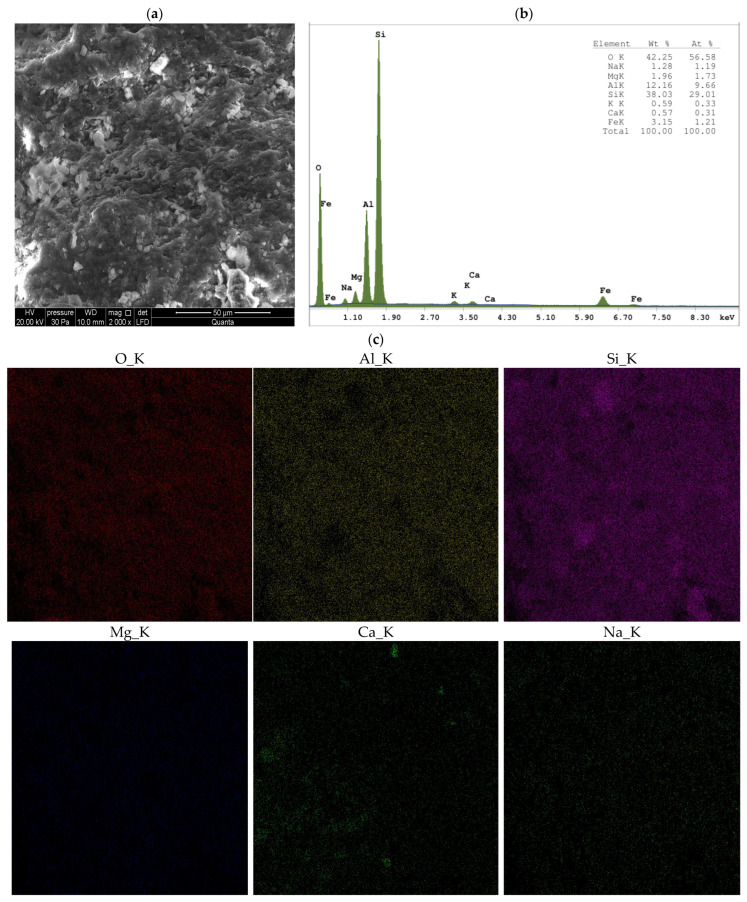
EDS Analysis of SWy-3 bentonite before treatment with ZnCl_2_: (**a**) area of analysis with microphotograph; (**b**) elemental composition spectrum; (**c**) spatial distribution of selected elements on the sample surface.

Energy-dispersive X-ray spectroscopy (EDS) provides localized, high-resolution elemental data from specific micro-areas (in our study, the analyzed surface area was approximately 150 μm × 150 μm), enabling spatial mapping of metal distribution and the identification of microscale heterogeneities in the elemental composition of materials [28,42]. EDS results for unmodified bentonites and for bentonites treated with ZnCl_2_ are presented in Figure A2, Figure A3 and Figure A4 and Figure 5, Figure 6 and Figure 7, respectively.

Surface analysis revealed that zinc and chlorine—the main components of simonkolleite—are distributed uniformly across the entire surface of the montmorillonite, including within the spaces between its aggregates (Figure 5, Figure 6 and Figure 7). This distribution, together with the XRD and SEM results, confirms the formation of the simonkolleite phase in this region.

Table 1 shows the chemical composition of the bentonites before and after contamination with ZnCl_2_, determined by two independent methods: inductively coupled plasma optical emission spectrometry (ICP-OES) and X-ray fluorescence (XRF). ICP-OES measures the concentration of ions in extracts from bentonite solutions, while XRF analyzes the composition of the solid sample in the surface region. This enables a complementary assessment of elemental composition changes in the material and the effectiveness of the ion exchange process.

The results of ICP-OES and XRF analyses, combined with XRD data and interlayer spacing measurements, indicate that ion exchange occurred in sodium and calcium bentonites. Quantitative ICP-OES analysis revealed that the calcium content in Ca-bentonites decreased nearly fourfold (BSvk: from 11,945 ± 140 to 2778 ± 31 mg/kg dm; STx-1b: from 11,802 ± 101 to 2985 ± 23 mg/kg dm), while the sodium content in Na-bentonite SWy-3 decreased approximately tenfold (from 10,086 ± 81 to 995 ± 9 mg/kg dm) after saturation with ZnCl_2_. In contrast, the zinc content significantly increased in all Zn-saturated bentonites (BSvk: from 64.54 ± 0.69 to 17,857 ± 89 mg/kg dm; STx-1b: from 73.68 ± 0.27 to 16,153 ± 75 mg/kg dm; SWy-3: from 163.66 ± 1.50 to 44,463 ± 124 mg/kg dm). These substantial increases in Zn concentration—considering that simonkolleite constitutes only about 30% of the sample volume (XRD, Figure 1)—strongly suggest that a significant portion of the metal is most likely located within the interlayer spaces of the montmorillonite. This hypothesis is further supported by the XRF results (Table 1), as well as by the interlayer spacing data discussed in Section 3.1.1.

The XRF suggests that the contamination of bentonites with ZnCl_2_ leads to structural changes in Mnt. As a result of ion exchange with Zn, the concentrations of Si, Al, Mg, and Fe decreased in each of the studied bentonites, which may indicate changes in the tetrahedral and octahedral layers of Mnt. Furthermore, the reduced concentrations of Ca, Mg, Na, and K, along with the simultaneous increase in Zn ion concentration, suggest competition between these ions and their replacement by Zn. The presence of Zn and Cl indicates a new mineral phase, identified by XRD as Skl. The concentrations of heavy metals are low and do not show significant changes after contamination of the bentonites with ZnCl_2_. A slight decrease in Mn, Ni, and Cu may indicate replacement by Zn.

A 1 molar (1 M) concentration of ZnCl_2_ allows for an effective ion exchange process, replacing Ca and Na ions with Zn in Na- and Ca-bentonites. Previous studies have shown that concentrations of approximately 0.004 M ZnCl_2_ did not allow for full ion exchange in bentonites, and the formation of Skl was not observed [13]. In contrast, Oueslati et al. [26] when saturating bentonites with 0.5 M ZnCl_2_, noticed the appearance of Skl. They did not evaluate the completeness of the ion exchange or the quantity of the newly formed mineral phase. Previous studies also indicate that the effectiveness of ion exchange in bentonites depends not only on the molar concentration but also on the type of exchangeable cation. In the case of CuCl_2_-contaminated bentonites, the exchange of Ca ions for Cu was fully effective at molar concentrations of 0.10–0.25–0.50 M, while at a concentration of 1 M, not all Ca ions were replaced by Cu [43].

### 3.3. Physical Analysis

Table 2 shows how the granulometric, sorption, and plastic properties of bentonites change after saturation with ZnCl_2_.

#### 3.3.1. Granulometric Composition, Plastic Parameters, and Soil Type

In ZnCl_2_-treated bentonites, a decrease in the clay fraction content (CLY), an increase in silt (SIL) and sand fraction content (SA), and an increase in the effective particle size (d_10_) (Table 2) were observed. The results may suggest the formation of aggregates. Tang et al. [47] discussed how aggregates may form and break down as a result of complex processes, including clay flocculation, swelling, dispersion, and salt crystallization in the soil. Angon et al. [48] confirm that Zn influences soil texture by altering the proportions of SA, SIL, and CLY. The reorganization of fractions ultimately leads to a change in soil classification (Table 2). For example, according to the United States Department of Agriculture (USDA) classification [44], before treatment, the bentonites are classified as silty loam (BSvk, STx-1b) or clay loam (SWy-3), while after treatment with ZnCl_2_, all bentonites are classified as silt in this system. Considering the USDA classification, the most significant hydrological changes occurred in the SWy-3 bentonite. This is concerning, as Na-bentonites are often used as geotechnical barriers [49]. Initially, bentonite was classified in category D as a material with a very low infiltration rate. After treatment with ZnCl_2_, it moved to category B as a material that is moderately to well permeable. Similarly, according to the classification by the EN-ISO 14688-1 standard [45], ZnCl_2_-treated bentonites are considered materials with poorer engineering properties, which affects their effectiveness as sealing and contaminant-absorbing materials.

Contamination of bentonites with 1M ZnCl_2_ led to a shift in the chemical equilibrium, and the pH changed from alkaline (~7.9) to neutral (~6.7). Nartowska et al. [19] observed a pH decrease from 7.21 to 6.09 in clay contaminated with 0.08 M ZnCl_2_. A similar pH decrease, despite a significantly higher salt concentration, may suggest that bentonites exhibit some resistance to drastic pH changes. However, even a slight reduction in pH to neutral levels may increase metal availability [50]. Previous studies by Nartowska et al. [17] showed that bentonites contaminated with ZnCl_2_ contain as much as 49.9–61.6% of potentially mobile fractions, with pH change being one of the contributing factors.

In bentonites treated with ZnCl_2_, a decrease in the liquid limit (LL) and an increase in the plastic limit (PL) is observed. The change in PL is most likely related to a reduction in intermolecular forces caused by a change in surface charge due to interactions with Zn ions. This effect results in a more stable structure, leading to smaller pores and reduced water availability, lowering the LL. The LL decreases up to fivefold in Na-bentonite, while in Ca-bentonite, the reduction is around 30%. Stronger changes in Na-bentonites are likely due to the smaller ionic radius of Na (0.95 Å) compared to Ca (1.0 Å), as well as weaker interactions with the bentonite particles, making Na-bentonites more susceptible to exchange with Zn ions. Ca-bentonites are promising, as the increase in the PL can enhance the flexibility and the ability of the bentonite to retain its properties under changing moisture conditions, providing better sealing. On the other hand, the decrease in the LL reduces excessive swelling, which is important to avoid excessive settlement.

#### 3.3.2. Specific Surface Area (SSA) and Porosity

SSA and porosity are key in assessing the interaction of clay particles with water and ZnCl_2_. SSA_WST_ is the total area of clay particles, and thus pores, available for this interaction (m^2^/g). SSA_BET_ measures the surface area, which includes both micropores (>0.7 nm) and mesopores (ranging from 2 to 50 nm in diameter). On the other hand, the SSA_BJH_ method covers the surface area of mesopores. Soil porosity determines how water and contaminants will move.

**Figure 8:**
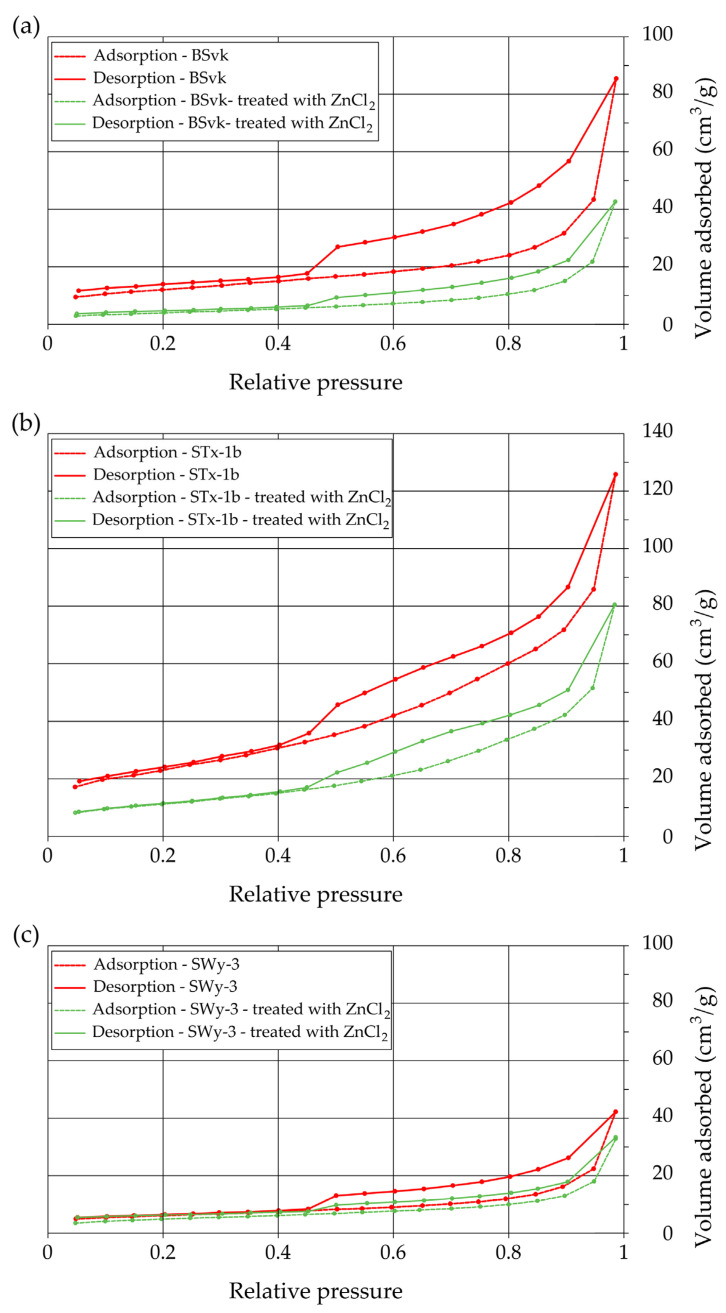
Nitrogen sorption/desorption isotherms for bentonites before and after ZnCl_2_ treatment: (**a**) calcium bentonite from Jelsový Potok, Slovakia (BSvk); (**b**) calcium bentonite from Texas, USA (STx1b); (**c**) sodium bentonite from Wyoming, USA (SWy-3).

**Figure 9:**
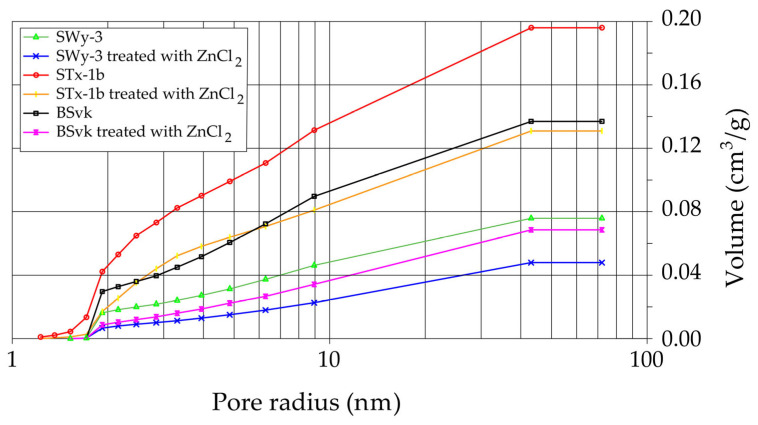
Total porosity of bentonites before and after treatment with ZnCl_2_ calculated using BJH method.

Figure 8 shows the nitrogen sorption/desorption isotherms for bentonites before and after ZnCl_2_ treatments. The results show that adsorption–desorption in bentonites contaminated with ZnCl_2_ is less effective compared to uncontaminated bentonites, likely due to reduced porosity (Figure 9) and changes in the surface properties of the bentonite caused by the interaction with Zn ions (Table 2). Similarly, Angon et al. [48] stated that high concentrations of potentially toxic metals (including Zn) can reduce soil porosity and pore connectivity, leading to decreased water-holding capacity and increased water runoff.

In Figure 8, adsorption–desorption hysteresis is observed in the higher relative pressure range (p/p0 > 0.4) for both contaminated and uncontaminated bentonites. This phenomenon is typical of porous materials and indicates the occurrence of capillary condensation [51]. High pressure can promote the condensation of molecules in micropores, hindering their removal during desorption, which leads to hysteresis in this range.

The SSA_BET_ results for SWy-3 (23.30 m^2^/g) and STx-1b (82.06 m^2^/g) are consistent with those reported by Byun et al. [52], who obtained slightly higher values: SWy-3 (28 m^2^/g) and STx-1b (93.2 m^2^/g). The minor differences are attributed to the different sample degassing temperatures used (105 °C in our study and 130 °C by Byun et al. [52]), as higher degassing temperatures generally remove more adsorbed water molecules and volatile substances, leading to slightly higher measured surface areas. The contamination of bentonite with ZnCl_2_ leads to a reduction in the specific surface area (SSA_BET_) and the surface area of mesopores (SA_BJH_) (Table 2), suggesting that contamination may block or alter the pore structure of bentonite, decreasing the availability of adsorption sites. Possible mechanisms include particle aggregation and reduced access to micropores (Table 2, an increase in d_10_), as well as the blocking of micropores in Mnt by Skl. This assumption may be supported by the higher SA_BJH_ values compared to SSA_BET_, which indicates a higher proportion of mesopores in adsorption, as also shown in Figure 8. A similar reduction in SSA_WST_ after ZnCl_2_ contamination is observed in Ca-bentonites (Table 2). However, in Na-bentonite (SWy-3), an increase in SSA_WST_ is visible. This can be explained by the fact that SSA_WST_ also includes the internal surface of the bentonite, which is unavailable to nitrogen, including interlayer spaces, whose distance increased after contamination of SWy-3 (11.44 Å to 13.2 Å) [17]. It is particularly noticeable in Na-bentonites, where a significant portion of Zn or Skl accumulates in the interlayer space. Similarly, studies by Hsiao et al. [25] demonstrated that the sodium silicate electrolyte in a zinc battery exhibited an increased specific surface area after HCl treatment. The increased SSA in Na-bentonites may enhance their ability to adsorb contaminants, which is beneficial for improving soil quality and removing toxic substances from groundwater. Such changes could increase the effectiveness of bentonites as protective barriers against contaminant migration; however, Skl may be less resistant than Mnt to the release of metals. Studies by Yamamoto et al. [53] confirm that Skl can gradually release Zn even when its structure is not modified.

The following summary explains the mechanisms that occur in bentonites contaminated with ZnCl_2_, detailing the processes at the mineralogical and physicochemical levels:

1. When Ca- or Na-bentonite with a pH of 7.92 ± 0.12 is treated with 1 M ZnCl_2_ (pH = 4.51 ± 0.01), the solution predominantly contains zinc ions (Zn^2+^) and chloride ions (Cl^−^). This treatment initiates an almost complete ion exchange process, in which Zn^2+^ ions replace Ca^2+^ or Na^+^ ions within the montmorillonite (Mnt) structure. As a result, the concentrations of Si, Al, Mg, and Fe decrease in all the studied bentonites. XRF analysis confirms that the content of Ca or Na approaches zero, indicating that the tetrahedral and octahedral layers of Mnt have undergone significant structural alterations. Unstable Mnt phases disappear in the XRD analysis.
2. In addition to the structural changes in montmorillonite (Mnt), a new mineral phase, simonkolleite (Skl), forms during the contamination process. Chemical reactions take place primarily at the montmorillonite phase boundary or within its structure, where the local pH is higher. EDS analysis confirms elevated levels of Zn and Cl^−^ in the contaminated bentonites, accompanied by a near-complete depletion of Ca or Na. This chemical transformation is a key indicator of Skl formation and has significant implications for the material’s physical properties, particularly the clay’s surface area and porosity.
3. Upon lowering the pH to 6.78 ± 0.05, SEM imaging reveals the characteristic needle-like morphology and hexagonal platelets of simonkolleite (Skl), confirming the formation of this new mineral phase. XRD analysis further supports its presence, indicating that approximately 30% of the contaminated bentonite consists of Skl.
4. Thermal analysis (TGA) confirms the presence of simonkolleite (Skl) and reveals thermal alterations in the montmorillonite (Mnt) structure resulting from physicochemical changes induced by ZnCl_2_ contamination. In particular, Zn^2+^ ions replacing Na^+^ in Na-bentonite accumulate within the interlayer spaces of Mnt, increasing the interlayer spacing and thereby enhancing the specific surface area for water (SSA_WST_). These structural modifications facilitate more efficient water flow through the material.
5. In Ca-bentonite, Zn replacing Ca predominantly accumulates on the surface of Mnt particles, leading to a decrease in specific surface area (SSA_BET_) and a reduction in surface area of mesopores (SA_BJH_), and porosity. This reduction is also observed in Na-bentonite. Possible mechanisms contributing to these changes include particle aggregation, reduced access to micropores, and the blocking of micropores in Mnt by Skl. This hypothesis is supported by the higher SA_BJH_ values than SSA_BET_, suggesting a higher proportion of mesopores in sorption. The aggregation, increasing effective diameter (d_10_), driven by processes like flocculation, swelling, dispersion, and salt crystallization, further reduces the ability of the material to adsorb water in micropores, which can increase its permeability.

Based on the observed mineralogical and physicochemical transformations induced by ZnCl_2_ contamination—such as extensive ion exchange, structural degradation of montmorillonite, simonkolleite formation, and reduced surface area and porosity—we suggest that bentonite barriers exposed to metal-rich leachates may experience long-term performance degradation. These changes could result in increased permeability and reduced contaminant retention, which are critical parameters for barrier integrity.

Consequently, our results emphasize the need for careful geochemical compatibility assessments between bentonite and expected leachate compositions during repository design. Furthermore, regulatory guidelines may need to account for potential mineralogical transformations and include long-term durability testing under realistic chemical conditions. This could help ensure the continued effectiveness of bentonite-based barriers in isolating hazardous waste over extended timescales.

## 4. Conclusions

This study is the first to analyze the impact of zinc(II) chloride (ZnCl_2_) contamination on the chemical composition, structure, and porosity of Na- and Ca-bentonites, with a particular focus on the formation of simonkolleite (Skl). Experimental evidence shows that ZnCl_2_ contamination can significantly impair the physical and chemical properties of these materials by inducing the formation of secondary mineral phases. The novelty of this research lies in establishing a direct link between Zn-induced phase transformation and the deterioration of key functional properties, which is crucial for the long-term reliability of bentonite-based barriers in geotechnical applications.

The main findings of this study are as follows:

1. Contamination with 1 M ZnCl_2_ significantly alters the mineralogical composition and structural stability of bentonites, as evidenced by the formation of simonkolleite (~30%). These changes were confirmed by XRD, TGA, SEM, and complementary analytical techniques.
2. Ion exchange between Zn^2+^ and Ca^2+^/Na^+^ within the montmorillonite structure leads to a marked reduction in Si, Al, Mg, and Fe content, along with the near-complete depletion of Ca and Na, as demonstrated by XRF and EDS data. EDS surface mapping revealed a uniform distribution of zinc and chlorine across the entire montmorillonite surface, including inter-aggregate spaces, supporting the formation of the simonkolleite phase.
3. The extent of montmorillonite degradation depends on the specific type of bentonite and the montmorillonite phases present. BSvk and STx-1b bentonites (calcium forms) appear to undergo more advanced structural damage than SWy-3 (sodium form), as evidenced by the complete disappearance of certain montmorillonite reflections.
4. The formation of Skl and the associated structural modifications cause a ~50% reduction in specific surface area and porosity, as determined by BET and BJH analyses, potentially compromising the material’s performance as a sealing barrier.

Future research should focus on the following:

1. Quantitative evaluation of hydraulic conductivity and mechanical strength in ZnCl_2_-contaminated bentonites;
2. Long-term stability assessments under variable environmental conditions;
3. Development of modified bentonite materials with improved resistance to potentially toxic metal-induced structural transformations.

## Figures and Tables

**Table 1:** Results of ICP-OES and XRF analysis for bentonites.

Chemical Analysis	Element	BSvk (Ca Form)	BSvk Treated with ZnCl_2_	STx-1b (Ca Form)	STx-1b Treated with ZnCl_2_	SWy-3 (Na Form)	SWy-3 Treated with ZnCl_2_
ICP-OES ^&^ [17]	[mg/kg dry mass]						
	Ca	11,945 ± 140	2778 ± 31	11,802 ± 101	2985 ± 23	8282 ± 57	4526 ± 34
	Na	1151 ± 9	1204 ± 22	1970 ± 18	885 ± 16	10,086 ± 81	995 ± 9
	Zn	64.54 ± 0.69	17,857 ± 89	73.68 ±0.27	16,153 ± 75	163.66 ± 1.50	44,463 ± 124
XRF *	Ca	1.14	0.02	1.33	0.03	1.02	0.04
	Mg	2.32	0.76	2.15	0.72	1.74	0.53
	K	0.19	0.09	0.14	0.06	0.42	0.18
	Na	0.17	−	0.31	−	1.22	−
	Al	8.42	3.68	7.16	2.85	8.31	3.39
	Si	24.29	11.38	37.20	15.17	27.14	11.73
	Fe	1.67	0.99	0.82	0.41	2.55	1.42
	Cl	−	2.16	−	2.15	−	2.88
XRF (potentially toxic metals)	Zn	−	21.22	−	21.24	−	21.01
	Mn	0.0511	0.0273	0.0134	0.0062	0.0148	0.0044
	Co	<0.0001	<0.0001	<0.0001	<0.0001	<0.0001	<0.0001
	Ni	0.0010	<0.0001	0.0009	<0.0001	0.0010	<0.0001
	Cu	0.0006	<0.0001	0.0005	<0.0001	0.0006	<0.0001
	Cd	<0.00005	<0.00002	<0.00005	<0.00002	<0.00002	<0.00002
	Pb	0.0028	0.0020	0.0012	0.0028	0.00367	0.00113

− below detection limits; ^&^ total content of major elements in the dry clay matrix determined with ICP-OES [17]; * standard deviation: Zn, Al ± 0.01; Cl ± 0.001; other elements < ±0.001.

**Table 2:** Physical properties of bentonites before and after treatments with ZnCl_2_.

Properties			BSvk	BSvk Treated with ZnCl_2_	STx-1b	STx-1b Treated with ZnCl_2_	SWy-3	SWy-3 Treated with ZnCl_2_
Soil classification ^$^	USDA		silt loam	silt	silt loam	silt	clay loam	silt
	EN-ISO 14688-1		clayey silt	silt	clayey silt	clayey silt	clay	silt
pH_KCl_(-) *			7.82	6.76	7.88	6.75	8.05	6.84
			alkaline	neutral	alkaline	neutral	alkaline	neutral
Granulometric **		CLY (%)	19.8	8.9	18.5	10.7	42.6	6.6
		SIL (%)	80.2	90.2	81.5	87	57.4	87
		SA (%)	0.0	0.9	0.0	2.3	0.3	6.4
		d_10_ (μm)	1.33	2.18	1.39	1.91	0.96	2.83
Plasticity ^§^		PL (%)	46	61	44	73	35	54
		LL (%)	165	119	142	101	519	104
Sorption/desorption ^#^		SA_BJH_ (m^2^/g)	58.80	22.19	96.27	57.21	31.24	15.33
		SSA_BET_ (m^2^/g)	41.39	14.38	82.06	40.55	23.30	17.16
		SSA_WST_ (m^2^/g)	671	557	568	538	307	516
		w_95_ (%)	30.14	20.10	29.08	22.67	21.67	17.05
Porosity ^ϵ^		total (cm^3^/g)	0.137	0.069	0.196	0.130	0.076	0.048
Pore radius		Dv (nm)	1.921	1.923	1.931	1.925	1.924	1.920

Notes: ^$^ the United States Department of Agriculture (USDA) [44], EN-ISO 14688-1 [45]; * EN ISO 10390:2021 [46]. The pH of the bentonites treated with ZnCl_2_ was determined after rinsing them to remove excess chloride ions. ** Laser diffraction method; CLY—clay (d ≤ 0.002 mm), SIL—silt (0.002 mm < d < 0.063 mm), SA—sand (0.063 mm < d < 2 mm), d_10_ (effective diameter—10% of the particles have a smaller diameter than d_10_) [17]. ^§^ Rolling test; Casagrande method acc. to EN ISO/TS 17892-12 [30]. ^#^ SSA—soil specific surface area. The Brunauer–Emmett–Teller (BET) method, the water vapor sorption test method (WST); SA—surface area of mesopore based on the Barrett–Joyner–Halenda (BJH) model (desorption); w_95_—the sorptive moisture at p/p0 = 0.95 [30].

## Data Availability

The original contributions presented in this study are included in the article. Further inquiries can be directed to the corresponding author.

## Abbreviations

The following abbreviations are used in this manuscript:

Mnt	Montmorillonite
Skl	Simonkolleite
SWy-3	Na-bentonite from Wyoming, USA
STx-1b	Ca-bentonite from Texas, USA
BSvk	Ca-bentonite from Jelsovy Potok, Slovakia
PTMs	Potentially toxic metals
SSA	Specific surface area of clay
SA	Surface area of mesopores
CLY	Clay fraction
SIL	Silt fraction
d_10_	Effective diameter—the particle diameter below which 10% of the mass is finer
XRD	X-ray diffraction
SEM	Scanning electron microscopy
TGA	Thermogravimetric analysis
XRF	X-ray fluorescence
EDS	Energy-Dispersive X-ray spectroscopy
BET	Brunauer–Emmett–Teller adsorption nitrogen method
BJH	Barrett–Joyner–Halenda method
WST	Water sorption test method

## Appendix A

**Table A1:** XRD-identified mineral phases in bentonites with PDF references.

Soil Type	Mineral Phase	Key	Chemical Formula	PDF Number *
BSvk	Montmorillonite	Mnt 1	(Na,Ca)_0.3_(Al,Mg)_2_Si_4_O_10_(OH)_2_·H_2_O	00-003-0015 **
		Mnt 2	NaMgAlSiO_2_(OH)·H_2_O	00-002-0014
	Quartz	Qz	SiO_2_	04-012-0490
	Biotite	Bt	K(Mg,Fe)_3_(AlSi_3_O_10_)(OH)_2_	01-086-2115
STx-1b	Montmorillonite	Mnt 1	(Na,Ca)_0.3_(Al,Mg)_2_Si_4_O_10_(OH)_2_·xH_2_O	00-003-0015 **
		Mnt 3	Na-Al-Si-O-OH-H_2_O	00-003-0019
		Mnt 4	15A Ca_0.2_(Al,Mg)_2_Si_4_O_10_(OH)_2·_4H_2_O	00-013-0135
		Mnt 5	CaMg_2_AlSi_4_(OH)_2·_H_2_O	00-002-0239
	Opal CT	Opal CT	SiO_2_	00-061-0035
	Quartz	Qz	SiO_2_	01-085-0457
SWy-3	Montmorillonite	Mnt 6	22A Na_0.3_(Al,Mg)_2_ Si_4_O_10_(OH)_2_·H_2_O	00-029-1499 **
		Mnt 7	(Al(OH)_2_)_0.33_Al_2_(Si_3.67_O_10_)·8H_2_O	00-011-0303
		Mnt 3	Na-Al-Si-O-OH-H_2_O	00-003-0019
	Quartz	Qz 1	SiO_2_	01-085-0865
		Qz 2	SiO_2_	01-070-2538
BSvk treated with ZnCl_2_	Montmorillonite	Mnt 1	(Na,Ca)_0.3_(Al,Mg)_2_Si_4_O_10_(OH)_2_·xH_2_O	00-003-0015 **
	Simonkolleite	Skl	Zn_5_(OH)_8_Cl_2_·H_2_O	01-077-2311
	Quartz	Qz	SiO_2_	04-005-0490
	Biotite	Bt	K(Mg,Fe)_3_(AlSi_3_O_10_)(OH)_2_	01-086-2115
* STx-1b treated with ZnCl_2_	Montmorillonite	Mnt 1	(Na,Ca)_0.3_(Al,Mg)_2_Si_4_O_10_(OH)_2_·xH_2_O	00-003-0015 **
	Simonkolleite	Skl	Zn_5_(OH)_8_Cl_2_·H_2_O	01-077-2311
	Opal CT	Opal CT	SiO_2_	00-061-0035
	Quartz	Qz	SiO_2_	01-085-0457
SWy-3 treated with ZnCl_2_	Montmorillonite	Mnt 8	14A Na_0.3_(Al,Mg)_2_Si_4_O_10_(OH)_2_·xH_2_O	00-013-0259 **
		Mnt 9	AlSi_2_O_6_(OH)_2_	00-002-0037
	Simonkolleite	Skl	Zn_5_(OH)_8_Cl_2_·H_2_O	00-007-0155
	Quartz	Qz 3	SiO_2_	00-005-0490

* the PDF-4+ database of the International Center for Diffraction Data (ICDD, Newtown Square, PA, USA); ** the major phase of montmorillonite (Mnt) for which the interplanar distances d_001_ were calculated.
